# 

**Published:** 1877-07

**Authors:** 


### CONTENTS:

ARTICLE I.—Diseased Gums. By L. C Ingersoll, D. D. S.......... 97

ARTICLE II.—Disintegration, Arrestation and Prevention of Caries By Marshall H. Webb, D. D. S.......... 107

ARTICLE III—The Discovery of Anæsthesia (Continued.) By J. Marion Sims, M. D. 114

ARTICLE IV.—The Dangers of Chloroform.......... 121

ARTICLE V.—Tetanus. By N F. Brown, M. D.......... 125

ARTICLE VI.—The Literary Estimate of Medical Art.......... 127

ARTICLE VII.—Case of Exsection of Half of Inferior Maxillary; Reproduction of Bone. By Robert A. McLean, M. D.......... 129

ARTICLE VIII.—Michigan Dental Association.......... 131

ARTICLE IX.—Adænoid Tumor of Soft Palate. By W. W. Taylor, M. D.......... 133

ARTICLE X.—On Dyspepsia. By Dr. Farquharson.......... 134

ARTICLE XI.—Sub-periosteal Surgery.......... 135

### EDITORIAL, ETC.

The Dental Office & Laboratory.......... 136

Meetings of Dental Associations.......... 138

Donation from Prof. Henry S. Chase.......... 138

### OBITUARY.

Thos. T. De Graffenreid, D. D. S.......... 139

### MONTHLY SUMMARY.

Precautions in Using the Hypodermic Syringe.......... 139
Death from Chloroform.......... 140
Action of Anæsthetics on Muscles and Nerves.......... 140
Croton Chloral in Facial Neuralgia.......... 141
How to Deprive Iodine of its Stain.......... 141
Salicylic Acid and its Compounds.......... 141
Nelaton's Method of Resuscitation from Chloroform.......... 142
Death from Ten Grains of Chloral.......... 142
Medical Examinations in Scotland.......... 142
Chlorate of Potash and Mercurials.......... 143
Mode of Administering Chloroform.......... 143
How to Cure a Cold in the Head.......... 144
Death from Self-Administration of Chloroform.......... 144
An Antidote to Chloroform.......... 144
Method of Preventing Cloudiness on Exploring Mirrors.......... 144

